# The cardioprotective properties of *Persicaria maculosa* and *Citrus sinensis* extracts against doxorubicin-induced cardiotoxicity in mice

**DOI:** 10.22038/AJP.2024.24101

**Published:** 2024

**Authors:** Mohammad Mohammad Zaki, Ibrahim Helmi El-Sayed, Mamdouh Abdel-Mogib, Ashraf Abdel-Hameed El-Shehawy, Omali Youssef El-Khawaga

**Affiliations:** 1 *Department of Chemistry, Faculty of Science, University of Kafrelsheikh, Kafr El-Sheikh, Egypt*; 2 *Department of Chemistry, Faculty of Science, University of Mansoura, Mansoura, Egypt*

**Keywords:** Antioxidants, Doxorubicin, Oxidative stress, Toxicity, Nrf2, CBR1

## Abstract

**Objective:**

This study assessed the cardioprotective properties of *Persicaria maculosa *(PME) and *Citrus sinensis *(CME) hydro-methanolic extracts, besides *Citrus sinensis* aqueous extract (CWE) against doxorubicin (DOX)-induced cardiotoxicity.

**Materials and Methods:**

The extracts were characterized. Mice were divided into eight groups: control (saline), DOX, protected (injected with 200 mg/kg of PME, CWE or CME for 21 days, orally, and DOX), and extracts (PME, CWE or CME administration, orally, for 21 days). DOX was injected (5 mg/kg, ip) on days 8, 13 and 18 of the experiment. Cardiac tumor necrosis factor-alpha (*TNF-α*), nuclear factor (erythroid-derived 2)-like 2 (*Nrf2*) and carbonyl reductase 1 (*CBR1*) expression levels, besides superoxide dismutase, catalase, malondialdehyde, nitric oxide and total protein levels were evaluated. Serum lactate dehydrogenase, creatine phosphokinase cardiac isoenzyme, aspartate transaminase, cholesterol, triglycerides and creatinine levels, as well as the cardiac tissues were examined.

**Results:**

Comparing with the control, DOX considerably (p<0.01) up-regulated *TNF-α* expression, malondialdehyde, nitric oxide, cardiac enzymes, lipids and creatinine levels, while it significantly (p<0.01) down-regulated *Nrf2* and *CBR1*. Additionally, DOX interfered with antioxidant enzymes' activities (p<0.01). Conversely, protected groups showed a significant (p<0.01) amelioration of DOX-induced cardiotoxic effects.

**Conclusion:**

The current study provides a new understanding of *P. maculosa *and* C. sinensis *cardioprotective mechanisms*. *The extracts' cardioprotective effects may be due to their antioxidant activities, ability to maintain the redox homeostasis through regulation of important antioxidant genes and primary antioxidant enzymes, and capability to recover inflammatory cytokines and lipids levels. Noteworthy, the tested extracts showed no toxic changes on the normal mice.

## Introduction

Doxorubicin (DOX) is one of the most recommended anticancer anthracyclines, nevertheless, the induction of cardiotoxicity limits its medical usage (Li et al., 2022 ▶). Oxidative stress (OS), minerals' level change and mitochondrial dysregulation are the main mechanisms by which DOX causes cardiotoxicity (Wagh et al., 2022 ▶). Nuclear factor (erythroid-derived 2)-like 2 (*Nrf2*) and carbonyl reductase 1 (*CBR1*) are activated to maintain cellular redox homeostasis and overcome OS (Park et al., 2022 ▶). *Nrf2* controls a large number of downstream genes involved in OS balance, making it the main antioxidant regulator (Amirmostofian et al., 2023 ▶; Cheng et al., 2019 ▶; Zabihi et al., 2018 ▶). Therefore, *Nrf2* regulation plays an important role in OS prevention (Zhou et al., 2022 ▶). In numerous biological processes, *CBR1* as a member of the dehydrogenase/reductase family, catalyzes the reduction reaction of the active carbonyl substrates (Kwon et al., 2019 ▶). Another target of anthracyclines is the toxicity of vascular endothelial cells, where DOX impairs endothelial flexibility, tube formation and migratory capacity via dysregulation of nitric oxide (NO) level (Nebigil and Désaubry, 2018 ▶). DOX also activates cellular death and inflammatory mediators such as tumor necrosis factor-alpha (*TNF-α*) (Rawat et al., 2021 ▶).

Unwanted side effects of chemically created medications include weight gain, hypertension, stomach ulcers and insulin resistance (Hesari et al., 2021 ▶). In contrast, bioactive substances from medical herbs are main part of phytomedicine due to their wealth of health advantages, easy access and low cost without major negative consequences (Dianat et al., 2015 ▶; Shaban et al., 2022 ▶). For instance, *Persicaria maculosa* (Polygonaceae), contains a variety of bioactive compounds. Prior research on its biological activity revealed that it has antioxidant, anti-inflammatory, antibacterial, anti-tumor, anti-ulcerogenic and antileukemic activities (Hromádková et al., 2010 ▶). *Citrus sinensis* (Rutaceae) is one of the most widely consumed fruits worldwide. The *Citrus sinensis* peel contains potent food-additives and antioxidants (Razola-Díaz et al., 2021 ▶). 

The cardioprotective activity of* P. maculosa* has not been clarified before, and *C. sinensis* peel in the phytomedicine has not been fully validated due to a lack of reliable scientific evidence. Thus, the study aimed to investigate the protective properties of *P. maculosa* aerial part and *C. sinensis* peel extracts against DOX-induced cardiotoxicity in mice and explore their biological activities.

## Materials and Methods

**Plants materials**

Fresh *P. maculosa* flowering aerial parts and *C. sinensis* fruits were authenticated and obtained by Dr. Mohamed E. Mostafa at the Protection Research Institute-ARC in Giza, Egypt. The plants were cleaned by water and air-dried till used. Samples of the plants were stored at the herbarium of Mansoura University, Mansoura, Egypt. 

**Extracts preparation**

For preparation of *P. maculosa* (PME) and *C. sinensis* peel (CME) hydro-methanolic extracts, procedure of Justine et al. (2019) ▶ was utilized with a little modification. The plants were dried, ground and extracted at room temperature with 80% (v/v) methanol for 48 hr at a ratio of 1:5 (w/v), and then filtered by filter papers (Whatman, 150 mm diameters). According to Jo et al. (2021) ▶, the *C. sinensis* peel aqueous extract (CWE) was prepared. Briefly, the peel was decocted (350 g in 1.5 L water) for 15 min and the first extract was discarded. A second extraction of the peel took place in 1.9 L of water for 20 min. The extracts were concentrated by rotary evaporator, dried in the air, weighed and stored at -20°C until used.

**Extracts' phytochemicals identification**

Extracts' phytochemical constituents of glycosides, alkaloids, carbohydrates, terpenoids, tannins, flavonoids, anthraquinones and saponins were characterized (Balamurugan et al., 2019 ▶). 

**Extracts' total phenolic and flavonoid contents**

The Folin Ciocalteau test with gallic acid calibration curve was used to measure the total phenolic contents (Singleton and Rossi, 1965 ▶), while the total flavonoid contents were assessed by the method described by Sakanaka et al. (2005) ▶ using rutin standard curve.

**Extracts'** ***in-vitro*** **antioxidant activities**

**2,2-diphenyl-1-picrylhydrazyl radical assay**

By using the 2,2-diphenyl-1-picrylhydrazyl radical (DPPH) test, which mentioned by Aadesariya et al. (2017) ▶, the antioxidant properties of the extracts were assessed. In short, different concentrations of each extract were made (0.5, 1.25, 2.5 and 5 mg/ml), then 50 µl of each concentration was added to DPPH solution (250 µl, 0.1 mM) and the mixture was incubated for 30 min in darkness. The absorbance was measured in comparison to each corresponding blank sample at 517 nm. The DPPH inhibition (%) was calculated as follows (Jha et al., 2014 ▶):

$\mathrm{DPPH inhibition}\left(\%\right)=\left(\frac{A_{D}-A_{T}}{A_{D}}\right)\times 100$

where A_T_ is the DPPH absorbance following the addition of the sample versus the sample blank and A_D_ is the DPPH solution absorbance.

The extract concentrations that block 50% of the radical (IC_50_) and the antiradical power (ARP) were determined (Jha et al., 2014 ▶).

**2,2-azinobis-(3-ethylbenzothiazoline-6-sulphonate) method**

The 2,2-azinobis-(3-ethylbenzothiazoline-6-sulfonate (ABTS) technique, as published by Re et al. (1999) ▶, was used to estimate the antioxidant effects. ABTS solution (2.6 ml, 7 mM) and potassium persulfate solution (2.6 ml, 2.5 mM) were combined to create a fresh stock solution and incubated in the dark for 24 hr at room temperature. Using the AG lab spectrophotometer, the absorbance was adjusted to 0.7± 0.02 at 734 nm. Then, 900 µl of previously diluted ABTS stock solution was combined with 100 µl of each extract's 10% (w/v) methanolic solution, and the absorbance at 734 nm was instantly measured in comparison to the absorbance of each corresponding sample blank. The following formula was used to determine the extracts' antioxidant activities (Re et al., 1999): 

$\mathrm{ABTS inhibition}\left(\%\right)=\left(\frac{A-A_{S}}{A}\right)\times 100$

where A_S_ is the absorbance following the addition of the extract against the sample blank and A is the diluted ABTS absorbance.

**Animals**

Mice (male albinos) were purchased from the Nile-Company for Drugs and Chemicals Industry (Egypt) and housed in the animal house of the Medical Technology Center Alexandria-University (Egypt) with an average age of 9 weeks and body weight (BW) of 23 g. Water and typical mouse meal was freely available to the animals*.*

**Treatments**

The mice were divided into eight groups (n=6 per group) after a week of acclimatization as follows: Control (received three intraperitoneal (ip) injections of 500 µl normal saline on days 8, 13 and 18 of the experiment); DOX (the cardiotoxic group); PME, CWE and CME (the extracts groups, only administered with the extracts); and D+PME, D+CWE and D+CME (the protected groups, administered with extracts before and during DOX inoculation). DOX was given as three injections (500 µl, 5 mg/kg BW, ip) on days 8, 13 and 18 for a total dosage of 15 mg/kg BW. The animals that received the extracts were given 200 µl (200 mg/kg every day for 21 days, orally). On day 23, the mice were sacrificed following a 12-hr fasting. The dose of the extracts was detected by following the Organization for Economic Co-operation and Development (OECD) guideline (2008). According to Singal et al. (1995) ▶ instructions, the dosage and route of administration for DOX (Adricin®, DOX saline solution produced by Hikma Specialized Pharmaceuticals, Egypt) were used.

**Blood and tissue** **samples**

Under diethyl ether anesthesia, blood was drawn from an ocular vein and centrifuged, and the serum samples were stored at -20°C. After cervical dislocation, the heart of each animal was removed, washed by saline, weighed and divided into parts for the biochemical, molecular and histological studies.

**Body weight change percentage and the heart weight index**

The following equation was used to determine the heart weight index (HWI) (Mutavdzin et al., 2019 ▶): 

$\mathrm{HWI}=\frac{\mathrm{Heart weight}(\mathrm{mg})}{\mathrm{Body weight}(g)}$

The following formula was used to compute the body weight change (BWC) percentage (Mutavdzin et al., 2019 ▶): 

$\mathrm{BWC}\left(\%\right)=\left(\frac{\mathrm{Final weight}-\mathrm{Initial weight}}{\mathrm{initial weight}}\right)\times 100$

**Blood biochemical assays**

Using the kits purchased from Egy-Chem for the lab technology (Cairo, Egypt), the activities of creatine phosphokinase cardiac isoenzyme (CK-MB), aspartate transaminase (AST) and lactate dehydrogenase (LDH), as well as the level of creatinine were assessed. The levels of total cholesterol (TC) and triglycerides (TG) were measured using kits bought from the Egyptian Company for Biotechnology (Cairo, Egypt).

**Tissue biochemical assays**

The cardiac samples were sonicated to create 10% (w/v) homogenate in cooled sodium phosphate buffer (pH 7, 0.1 M) and centrifuged at 4000 rpm for 10 min at 4°C (Labofuge 400R cooling centrifuge). Each sample's supernatant was kept at -80°C until needed. Following a previous methodology outlined by Lowry et al. (1951), the total protein (TP) levels were estimated using a bovine serum albumin standard calibration curve. Nitric oxide (NO) and malondialdehyde (MDA) levels were measured using kits purchased from the Egyptian Company for Biotechnology (Cairo, Egypt). Using kits from the Biodiagnostic Company (Cairo, Egypt), the activities of the enzymes superoxide dismutase (SOD) and catalase (CAT) were tested.

**Molecular study**

Using an RNA isolation kit from Qiagen (Germany), the total RNA was extracted in accordance with the manufacturer's recommended procedure. Using an Applied Biosystems one-step instrument (Singapore), the expression levels of the cardiac *TNF-α*, *Nrf2* and *CBR1* genes were assessed in accordance with the instructions of the kit that bought from Enzynomics (Daejeon, Korea). In brief, the enzyme mixture (1 µl) was combined with the template (1 µl, 500 ng/µl), forward and reverse primers (1 µl each, 10 µM), SYBR mixture (10 µl) and the mixture was completed up to 20 µl by sterile RNase-free water. Using Glyceraldehyde-3-phosphate dehydrogenase (*GAPDH*) as a housekeeping gene, the fold expression was estimated using the comparative (CT) method (Livak and Schmittgen, 2001 ▶). The primer sequences for *Nrf2*, *CBR1* and *TNF-α* that were mentioned in previous studies (Guo et al., 2018 ▶; Olson et al., 2003 ▶; Wang et al., 2021 ▶), were created by SBS Genetech Company (Beijing, China) and displayed in Table 1.

**Histopathological study**

The fixed samples were dehydrated and stained with hematoxylin and eosin (Suzuki and Suzuki, 1998 ▶).

**Statistical analysis**

The mean and standard deviation (S.D.) of data are used in statistical descriptions. One-way ANOVA with Tukey's post hoc test was used for statistical analysis and a p˂0.05 was considered significant.

## Results

**Extracts characterizations**

**Figure 1:**
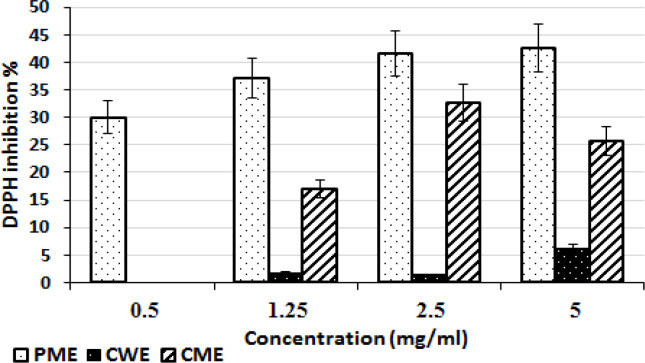
The extracts' DPPH inhibition (%) at various concentrations. (Means±S.D.), PME (*Persicaria maculosa* hydro-methanolic extract), CME (*Citrus sinensis* hydro-methanolic extract), CWE (*Citrus sinensis* aqueous extract) and DPPH (2,2-diphenyl-1-picrylhydrazyl radical)

The extracts properties are summarized in Figure 1, and Tables 2 and 3.

**Table 1:** The primers in RT-qPCR analysis

**Gene**	** Primers (5′- 3')**	
***Nrf2***	Forward	TAG TGC CCC TGG AAG TGT CA
	Reverse	TTG GGA TTC ACG CAT AGG AG
***CBR1***	Forward	GGT GCT AAC AAA GGA ATC
	Reverse	CTC TGC TTG AAT GTG GAA
***TNF-α***	Forward	ACT CAA CAA ACT GCC CTT CTG AG
	Reverse	TTA CAG CTG GTT TCG ATC CAT TT
***GAPDH***	Forward	TGC ATC CTG CAC CAC CAA CT
	Reverse	TGC CTG CTT CAC CAC CTT C

*Nrf2 *(Nuclear factor (erythroid-derived 2)-like 2), *CBR1* (Carbonyl reductase 1), *TNF-α* (Tumor necrosis factor-alpha), and *GAPDH* (Glyceraldehyde-3-phosphate dehydrogenase).

**Table 2:** Total phenolic and flavonoid contents, and antioxidant properties of the extracts

**Extract**	**Total phenols** **(µg GE/mg extract)**	**Total flavonoids** **(µg RE/mg extract)**	**ABTS inhibition (%)**	**IC**_50_ ** (mg/ml)**	**ARP**
**PME**	445.3±3.2	287.8±19.0	64.4±3.9	10.4±0.6	0.10±0.01
**CWE**	140.9±5.0	21.6±1.4	52.8±3.2	24.8±1.5	0.04±0.00
**CME**	132.5±1.6	9.5±0.6	56.9±3.4	9.4±0.6	0.11±0.01

Data are presented as mean±SD. PME (*Persicaria maculosa* hydro-methanolic extract), CME (*Citrus sinensis* hydro-methanolic extract), CWE (*Citrus sinensis* aqueous extract), GE (Gallic acid equivalent), RE (Rutin equivalent), IC_50_ (extract concentrations that block 50% of radicals) and ARP (Antiradical power).

**Table 3:** Extracts phytochemicals contents

**Phytochemical **	**Test**	**PME**	**CWE**	**CME**
**Alkaloids and/or nitrogenous bases**	Wagner's test	+	+	+
**Carbohydrates and/or glycosides**	Molisch's and Borntrager's tests	+	+	+
**Tannins**	Ferric chloride test	+	+	+
**Flavonoids**	General lab test	+	+	+
**Saponins**	Froth test	+	-	+
**Triterpenoids**	General lab test	+	+	+
**Anthraquinones**	General lab test	+	-	-

(+) detected, (-) not detected, PME (*Persicaria maculosa* hydro-methanolic extract), CME (*Citrus sinensis* hydro-methanolic extract) and CWE (*Citrus sinensis* aqueous extract).

**Extracts administrations with DOX restored the body's normal weight and heart weight index**

The body weight change percent (BWC %), heart weight index (HWI), and cardiac total protein (TP) were estimated to reflect the protective effects of the extracts against the DOX-induced toxic changes (Table 4). In comparison to the control group, administration of DOX caused a significant (p˂0.01) decrease in BWC % and increase in HWI. The protected groups displayed a substantial (p˂0.01) increase in BWC % and decrease in HWI when compared to the DOX group. The BWC% and HWI of the extracts groups did not differ significantly from the control group except the CWE group which showed a significant (p˂0.01) drop in BWC%. Cardiac TP data showed that all treatments were non-significantly different from the control group, with the exception of the D+CWE group that showed a significant (p˂0.01) increase in TP level. 

**The extracts protected cardiomyocytes from DOX-induced injury**

**Figure 2:**
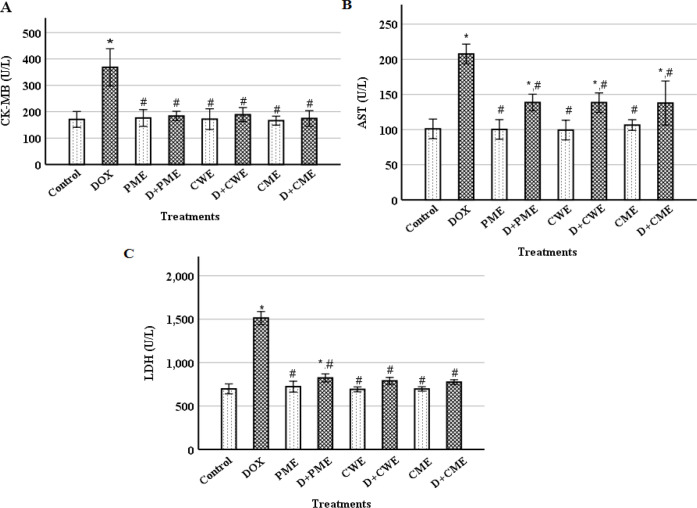
Effect of DOX and the extracts on creatine kinase cardiac isoenzyme (CK-MB) (A), lactate dehydrogenase (LDH) (B) and AST (aspartate transaminase) (C) activities. (Means±S.D., n=6). *(p<0.01) compared to the control group and ^#^(p<0.01) compared to the DOX group

The serum levels of CK-MB, LDH and AST were examined to clarify the cardiac tissue damage. The DOX group's cardiac CK-MB, LDH and AST levels showed significant rises compared to the control group (p˂0.01) (Figure 2). In the protected groups, compared with the DOX group, ameliorated measurements (p˂0.01) were noted. The extracts groups compared to the control, did not exhibit any significant changes.

**Table 4:** Changes in the body weight, heart weight index, and cardiac total protein

**Treatment**	**Body weight (g)** **Initial Final**		**BWC (%)**	**HWI**	**TP** **(mg/g tissue)**
**Control**	25.8±2.0	33.5±3.9	29.8±5.1	5.1±0.3	80.1±3.2
**DOX**	32.5±3.1	25.7±1.9	-20.9±1.7^*^	6.3±0.2^*^	82.9±5.8
**D+PME**	22.7±1.7	24.4±2.5	7.5±3.0^*, #^	5.1±0.4^#^	83.2±6.3
**PME**	26.6±2.0	32.6±1.9	22.6±2.1^#^	5.1±0.3^#^	86.9±7.6
**D+CWE**	29.0±1.9	27.9±1.3	-3.8±1.8^*, #^	5.0±0.2^#^	98.1±9.5^*^
**CWE**	27.9±2.2	33.2±3.1	19.0±1.7^*, #^	5.1±0.7^#^	90.3±7.1
**D+CME**	30.1±0.5	27.9±0.7	-7.3±0.5^*, #^	5.0±0.5^#^	85.4±10.2
**CME**	24.6±2.8	31.1±2.8	26.4±3.0^#^	5.0±0.6^#^	86.2±8.8

Data are presented as mean±SD, n=6. *(p<0.01) compared to the control group and ^#^(p<0.01) compared to the DOX group. BWC% (Body weight change percent, the negative (-) value means a decrease and the positive (+) means an increase), HWI (Heart weight index) and TP (Total protein).

**The oxidative status of the cardiomyocytes was improved by the extracts**

**Figure 3:**
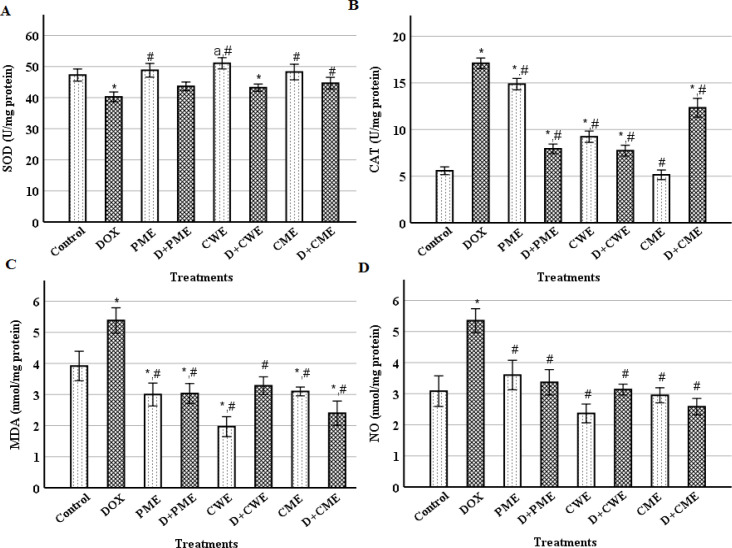
Effect of treatment with DOX and/or extracts on superoxide dismutase (SOD) (A), catalase (CAT) (B), malondialdehyde (MDA) (C) and nitric oxide (NO) (D) levels. (Means±SD., n=6). ^* ^(p<0.01), ^a^ (p<0.05) as compared to the control, and ^#^ (p<0.01) as compared to the DOX group

We investigated the OS indicators and enzymatic antioxidant activity in the heart tissue. Following DOX injection, SOD activity was significantly (p˂0.01) reduced, but CAT activity, and MDA and NO levels were significantly (p˂0.01) elevated as compared to the control values. In contrast to the DOX group, the protected groups had considerably (p˂0.01) lower CAT activity, and MDA and NO levels. Additionally, the protected groups' SOD activity slightly increased compared to the DOX group (Figure 3). Comparing with the control, SOD activity of the extracts groups was non-significantly increased, except for the CWE group that showed a significant (p˂0.05) increase in SOD activity. In addition, the CAT activity was significantly (p˂0.01) increased in the PME and CWE groups, but it non-significantly increased in the CME group compared to the control group. MDA levels in the extracts groups were significantly (p˂0.01) reduced as compared to the control, and the NO level showed non-significant changes (Figure 3).

**Extracts administrations with DOX regulated** ***Nrf2*** **,** ***CBR1*** **and** ***TNF-α*** **expression levels**

**Figure 4:**
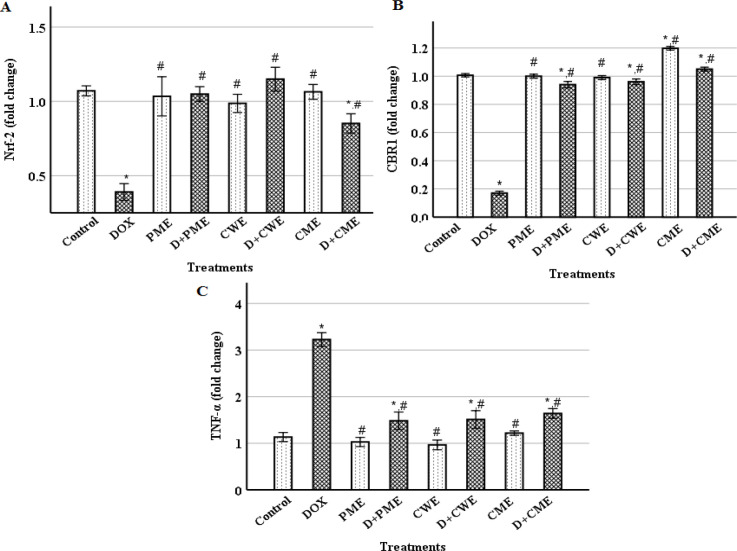
Cardiac nuclear factor (erythroid-derived 2)-like 2* (Nrf2*) (A), carbonyl reductase 1(*CBR1*) (B) and tumor necrosis factor-alpha (*TNF-α*) (C) expression levels. (Means±S.D., n=6). *(p<0.01) compared to the control group and ^#^ (p<0.01) compared to the DOX group

The mRNA levels of *Nrf2*, *CBR1* and *TNF-*α in the myocardium were examined using the RT-qPCR technology in order to explore the molecular effects. Figure 4 shows that DOX injection considerably (p˂0.01) decreased *Nrf2* and *CBR1* expression levels, while it significantly (p˂0.01) increased *TNF-α* expression level in comparison to the control. In contrast to the DOX group, the *TNF-α* expression was decreased, but *Nrf2* and *CBR1* expression levels were considerably (p˂0.01) elevated in the protected groups. The extracts groups, as compared with the control, showed non-significant changes in these genes, except CME group that showed a significant (p˂0.01) elevation in *CBR1* level.

**Extracts treatments with DOX reduced abnormalities in serum lipids and creatinine levels**

**Figure 5:**
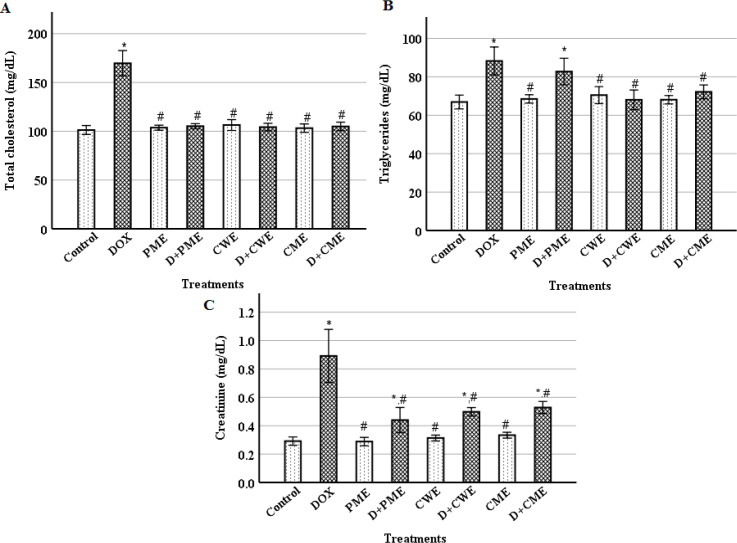
. Levels of serum total cholesterol (A), triglycerides (B), and creatinine (C). (Means±S.D., n=6). *(p<0.01) compared to the control group and ^#^(p<0.01) compared to the DOX group

By measuring the amounts of TC, TG and creatinine, it was possible to determine how DOX and the extracts affected serum lipids and kidney damage. In comparison to the control, the DOX group's TC, TG and creatinine levels were significantly (p˂0.01) elevated. Comparing with the DOX group, the TC, TG and creatinine levels of the protected groups were significantly (p˂0.01) decreased, except TG level of the D+PME group that showed a non-significant decrease. The levels of TC, TG and creatinine in the extracts groups showed non-significant differences compared with the control (Figure 5). 

**Extracts ameliorated DOX-induced heart's histological alterations**

**Figure 6:**
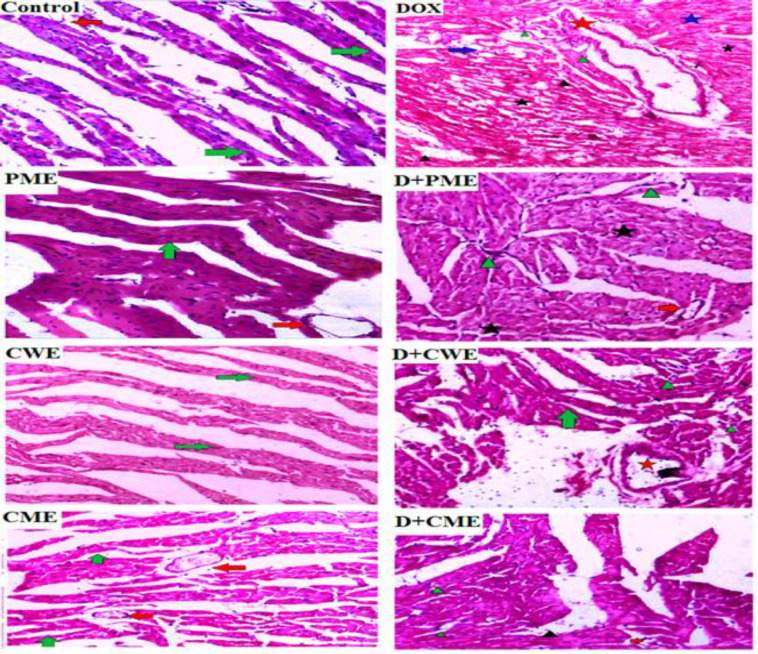
Influence of different treatments on the heart tissue (H&E staining, 20X magnification). The myocardial fibers in the control image are normal in appearance and arrangement (green arrow), and a typical blood vessel (red arrow) is shown with them. DOX, on the other hand, shows vacuolated myocardial fibers with the disordered arrangement (blue arrow), an obviously thickened blood vessel with an elongated wall (red star), hyperemia (black arrow-head), infiltration of fibroblasts and lymphatic cells (green arrow-head); D+PME, it identify a regular blood vessel (red arrow), a little amount of fibroblast infiltration (green arrowhead), and minor myocytolysis (black star); PME demonstrates the typical appearance of the myocardial fibers (green arrow) with regular blood vessels (red arrow); Normal cardiac fibers are revealed by D+CWE (green arrow), along with a very little amount of inflammatory cells (green arrowhead), and a mildly dilated blood vessel with a thicker wall (red star). CWE shows a blood vessel with a typical appearance (red arrow), D+CME shows a blood vessel with a typical appearance (red arrow), mild lymphatic and fibroblast infiltration (green arrowhead), and hyperemia (black arrowhead), and CME shows the normal myocardial fibers appearance (green arrow) with healthy blood vessel (red arrow)

We studied the histological alterations in heart tissue to support our theory. Figure 6 shows the myocardial fibres and blood vessels of the control and the extracts groups in their typical configuration. While DOX injection caused histopathological changes that were primarily evident in the disorganized arrangement of myocardial tissue with vacuolation, blood vessels with visibly thickened and elongated walls, hyperemia, excessive fibroblast and lymphatic cell infiltration, significant cardiocytes hyperplasia, and myocytolysis. The protected group, on the other hand, displayed markedly ameliorated histological alterations against DOX-induced toxicity.

## Discussion

DOX, a cancer treatment medication, has been linked to organs toxicity that leads to therapy termination (Li et al., 2022 ▶). To mitigate this issue, efforts are being made to reduce DOX-induced toxicity. *Persicaria maculosa* and *Citrus sinensis* are rich sources of anti-inflammatory and antioxidant compounds (Hromádková et al., 2010 ▶; Razola-Díaz et al., 2021 ▶). 

Our study demonstrates cardioprotective effects of *P. maculosa* aerial part hydro-methanolic extract and *C. sinensis* peel hydro-methanolic and aqueous extracts through physiological, biochemical, molecular and histological examinations. The DOX injection significantly decreased body weight due to its deleterious effects on food intake, nutrient absorption and protein synthesis (Elblehi et al., 2021 ▶). In line with a prior work, current study found that DOX administration resulted in cardiac hypertrophy in mice, as evidenced by a higher HWI compared to the control. (Mutavdzin et al., 2019 ▶). Our results confirmed the cardiomyocytes injury through the significant increases in cardiac enzymes after DOX injection compared to the control, supporting earlier findings (Satyam et al., 2023 ▶). DOX damages cardiomyocytes through reduction of DOX quinone moiety to produce semiquinone which consequently reacts with oxygen to produce superoxide radicals that induces the OS (Damiani et al., 2016 ▶; Doroshow et al., 2020 ▶). The DOX accumulation in the mitochondria disturbs the electron-transport chain and triggers the generation of reactive species (Qi et al., 2022 ▶). Additionally, the non-enzymatic reduction of Fe^3+^ by DOX results in the creation of DOX-Fe^2+^ radical complexes which disrupt cellular function and membrane integration (Rochette et al., 2015 ▶). Consequently, the high radicals levels trigger cytotoxic signals that cause biomolecule denaturation and cardiomyocytes death which increases the activity of cardiac enzymes in the serum (Rochette et al., 2015 ▶).

The decrease in SOD activity after DOX injection may be attributed to the oxidation of SOD functional residues and the consumption of the enzyme during OS reduction (Sandamali et al., 2019 ▶; Shaban et al., 2022 ▶). The increased CAT activity following DOX treatment may be a response to the DOX-induced oxidative circumstances that were produced from excessive production of the free radicals especially H_2_O_2_. These findings are consistent with earlier findings (Hadi et al., 2021 ▶; López-Barrera et al., 2020 ▶; Yin et al., 1998 ▶) . *Nrf2* gene is crucial for maintaining cellular redox balance by inhibiting OS and lipid peroxidation (LPO) through increasing the expression of antioxidant genes like *SOD* and *CBR1* (Cheng et al., 2019 ▶; Kwon et al., 2019 ▶). CBR1 is a dehydrogenase/reductase that prevents the OS and its cellular environment toxicity by reducing active carbonyl substrates (Kwon et al., 2019 ▶). Our molecular studies indicate that DOX injection decreases cardiac *Nrf2* and *CBR1* expression compared to the control as previously reported (Al-Kenany and Al-Shawi, 2023 ▶; Sharma et al., 2020 ▶; Zhang et al., 2021 ▶). The observed rise in cardiac *TNF-α* expression was attributed to the production of OS and LPO products, which are directly linked to cardiac pathogenesis (Akinloye et al., 2023 ▶; Nebigil and Désaubry, 2018 ▶; Zhang et al., 2019 ▶). The current study indicates that DOX administration significantly increased cardiac NO levels, indicating its potential toxicity to vascular endothelial cells (Nebigil and Désaubry, 2018 ▶). Additionally, in the present results DOX impaired renal functions and lipids metabolism as previously revealed (Afsar et al., 2020 ▶; Tan et al., 2011 ▶).

On the other hand, the protected groups showed significant increases in body weight, and decreases in HWI and cardiac enzymes, and the extracts effectively prevented disruptions in antioxidant enzyme activities and lipids, increased cardiac *Nrf2* and *CBR1* expressions and SOD activity, but the MDA and NO levels were decreased, indicating that the extracts prevented heart hypertrophy and damage, enhanced cardiac functions, and reduced the oxidative damage (Zhang et al., 2021 ▶). Similar to our findings, Kwon et al. (2019) ▶ showed that sulforaphane, an *Nrf2* activator, can reduce free radical formation and prevent LPO in hepatic cell lines. Furthermore, the protected groups showed reduction in cardiac *TNF-α* expression, cellular-oxidative damage and inflammatory conditions compared to the DOX group (Zhang et al., 2019 ▶). Previously it was shown that the extracts are rich in flavonoids, tannins, alkaloids and triterpenoids that possess significant antioxidant properties and crucial for heart protection (Omoba et al., 2015 ▶; Quesada-Romero et al., 2020 ▶). Our microscopic investigation showed the cardiomyocytes inflammation and degenerative changes after DOX injection in line with Razmaraii et al. (2016) ▶ results, but the protective treatments halted the DOX-induced toxicity. However, it is better to compare the effects of the extracts with the effects of standard cardioprotective agents in future investigations.

The current study revealed for the first time the cardioprotective mechanisms of the hydro-methanolic extracts of *Persicaria maculosa* and *Citrus sinensis* peel and the aqueous extract of *Citrus sinensis* peel against DOX-induced cardiotoxicity in mice. The extracts cardioprotective properties have been proved through enhancement of cardiac functions and prevention of the DOX-induced histopathological changes. The protective effects of the extracts may be attributed to their ability to regulate the cellular redox homeostasis, and hypolipidemic and anti-inflammatory properties. Overall, the tested extracts exhibited non-significant differences in their cardioprotective potentialities when compared with each other. Of note, the tested extracts showed no toxic effects on the normal animals, so *P. maculosa* and *C. sinensis* are effective as anticardiotoxic and can be considered good natural sources of cardioprotective constituents.

## Conflicts of interest

The authors have declared that there is no conflict of interest.

## References

[B1] Aadesariya M, Ram V, Dave P (2017). Evaluation of antioxidant activities by use of various extracts from Abutilon Pannosum and Grewia Tenax in the Kachchh region. MOJ Food Process Technol.

[B2] Afsar T, Razak S, Almajwal A, Al-Disi D (2020). Doxorubicin-induced alterations in kidney functioning, oxidative stress, DNA damage, and renal tissue morphology; Improvement by Acacia hydaspica tannin-rich ethyl acetate fraction. Saudi J Biol Sci.

[B3] Akinloye O, Sulaimon L, Ogunbiyi O, Odubiyi A, Adewale A, Toriola M, Salami O, Boyenle I (2023). Amaranthus spinosus (Spiny Pigweed) methanol leaf extract alleviates oxidative and inflammation induced by doxorubicin in male sprague dawley rats. Adv Trad Med.

[B4] Al-Kenany SA, Al-Shawi NN (2023). Protective effect of cafestol against doxorubicin-induced cardiotoxicity in rats by activating the Nrf2 pathway. Front Pharmacol.

[B5] Amirmostofian M, Akbari F, Hashemzaei M, Safaeinejad F, Tabrizian K, Arbab H, Rezaee R, Hemat S (2023). Hormetic effects of curcumin on oxidative stress injury induced by trivalent arsenic in isolated rat hepatocytes. Avicenna J Phytomed.

[B6] Balamurugan V, Fatima S, Velurajan S (2019). A guide to phytochemical analysis. Int J Adv Res Inn Ideas In Ed.

[B7] Cheng D, Gao L, Su S, Sargsyan D, Wu R, Raskin I, Kong AN (2019). Moringa isothiocyanate activates Nrf2: potential role in diabetic nephropathy. AAPS J.

[B8] Damiani RM, Moura DJ, Viau CM, Caceres RA, Henriques JAP, Saffi J (2016). Pathways of cardiac toxicity: comparison between chemotherapeutic drugs doxorubicin and mitoxantrone. Arch Toxicol.

[B9] Dianat M, Veisi A, Ahangarpour A, Moghaddam HF (2015). The effect of hydro-alcoholic celery (Apiumgraveolens) leaf extract on cardiovascular parameters and lipid profile in animal model of hypertension induced by fructose. Avicenna J Phytomed.

[B10] Doroshow JH, Esworthy RS, Chu FF (2020). Control of doxorubicin-induced, reactive oxygen-related apoptosis by glutathione peroxidase 1 in cardiac fibroblasts. Biochem Biophys Rep.

[B11] Elblehi SS, El-Sayed YS, Soliman MM, Shukry M (2021). Date Palm pollen extract avert doxorubicin-induced cardiomyopathy fibrosis and associated oxidative/nitrosative stress, inflammatory cascade, and apoptosis-targeting bax/bcl-2 and caspase-3 signaling pathways. Animals.

[B12] Guo Z, Yan M, Chen L, Fang P, Li Z, Wan Z, Cao S, Hou Z, Wei S, Li W (2018). Nrf2‑dependent antioxidant response mediated the protective effect of tanshinone IIA on doxorubicin‑induced cardiotoxicity. Exp Ther Med.

[B13] Hadi NA, Mahmood RI, Al-Saffar AZ (2021). Evaluation of antioxidant enzyme activity in doxorubicin treated breast cancer patients in Iraq: a molecular and cytotoxic study. Gene Reports.

[B14] Hesari M, Mohammadi P, Khademi F, Shackebaei D, Momtaz S, Moasefi N, Farzaei MH, Abdollahi M (2021). Current advances in the use of nanophytomedicine therapies for human cardiovascular diseases. Int J Nanomed.

[B15] Hromádková Z, Hirsch J, Ebringerová A (2010). Chemical evaluation of Fallopia species leaves and antioxidant properties of their non-cellulosic polysaccharides. Chemical Papers.

[B16] Jha DK, Panda L, Ramaiah S, Anbarasu A (2014). Evaluation and comparison of radical scavenging properties of solvent extracts from Justicia adhatoda leaf using DPPH assay. Appl Biochem Biotechnol.

[B17] Jo Y-J, Cho H-S, Chun J-Y (2021). Antioxidant activity of β-cyclodextrin inclusion complexes containing trans-cinnamaldehyde by DPPH, ABTS and FRAP. Food Sci Biotechnol.

[B18] Justine VT, Mustafa M, Kankara SS, Go R (2019). Effect of drying methods and extraction solvents on phenolic antioxidants and antioxidant activity of Scurrula ferruginea (Jack) Danser (Loranthaceae) leaf extracts. Sains Malaysiana.

[B19] Kwon JH, Lee J, Kim J, Kirchner VA, Jo YH, Miura T, Kim N, Song GW, Hwang S, Lee SG (2019). Upregulation of carbonyl reductase 1 by Nrf2 as a potential therapeutic intervention for ischemia/reperfusion injury during liver transplantation. Mol Cells.

[B20] Li C, Zhang L, Bu X, Wang J, Li L, Yang Z (2022). Circ-LTBP1 is involved in doxorubicin-induced intracellular toxicity in cardiomyocytes via miR-107/ADCY1 signal. Mol Cell Biochem.

[B21] Livak KJ, Schmittgen TD (2001). Analysis of relative gene expression data using real-time quantitative PCR and the 2− ΔΔCT method. Methods.

[B22] López-Barrera LD, Díaz-Torres R, Martínez-Rosas JR, Martínez AMS, Rosales C, Ramirez-Noguera P (2020). Chitosan-glutathione nanoparticles modify oxidative stress induced by doxorubicin in breast cancer cells. Beilstein Arch.

[B23] Lowry OH, Rosebrough NJ, Farr AL, Randall RJ (1951). Protein measurement with the Folin reagent. Biol Chem.

[B24] Mutavdzin S, Gopcevic K, Stankovic S, Jakovljevic Uzelac J, Labudovic Borovic M, Djuric D (2019). The effects of folic acid administration on cardiac oxidative stress and cardiovascular biomarkers in diabetic rats. Oxid Med Cell Longev.

[B25] Nebigil CG, Désaubry L (2018). Updates in anthracycline-mediated cardiotoxicity. Front Pharmacol.

[B26] OECD (2008). Test No. 425: Acute oral toxicity: Up-and-Down procedure.

[B27] Olson LE, Bedja D, Alvey SJ, Cardounel A, Gabrielson KL, Reeves RH (2003). Protection from doxorubicin-induced cardiac toxicity in mice with a null allele of carbonyl reductase 1. Cancer Res.

[B28] Omoba OS, Obafaye RO, Salawu SO, Boligon AA, Athayde ML (2015). HPLC-DAD phenolic characterization and antioxidant activities of ripe and unripe sweet orange peels. Antioxidants.

[B29] Park C, Lee H, Kim SO, Lee EW, Lee HT, Kwon HJ, Kim BW, Kim GY, Kim MR, Choi YH (2022). The preventive effect of Mori Ramulus on oxidative stress-induced cellular damage in skeletal L6 myoblasts through Nrf2-mediated activation of HO-1. Toxicol Res.

[B30] Qi Y, Chen J, Duan J, Kang L, Wang K, Chen Z, Xu B, Gu R (2022). Major vault protein attenuates cardiomyocyte injury in doxorubicin-induced cardiomyopathy through activating AKT. BMC Cardiovasc Disord.

[B31] Quesada-Romero L, Fernández-Galleguillos C, Bergmann J, Amorós M-E, Jiménez-Aspee F, González A, Simirgiotis M, Rossini C (2020). Phenolic fingerprinting, antioxidant, and deterrent potentials of Persicaria maculosa Extracts. Molecules.

[B32] Rawat PS, Jaiswal A, Khurana A, Bhatti JS, Navik U (2021). Doxorubicin-induced cardiotoxicity: An update on the molecular mechanism and novel therapeutic strategies for effective management. Biomed Pharmacother.

[B33] Razmaraii N, Babaei H, Nayebi AM, Assadnassab G, Helan JA, Azarmi Y (2016). Crocin treatment prevents doxorubicin-induced cardiotoxicity in rats. Life Sci.

[B34] Razola-Díaz MdC, Guerra-Hernández EJ, Rodríguez-Pérez C, Gómez-Caravaca AM, García-Villanova B, Verardo V (2021). Optimization of ultrasound-assisted extraction via sonotrode of phenolic compounds from orange by-products. Foods.

[B35] Re R, Pellegrini N, Proteggente A, Pannala A, Yang M, Rice-Evans C (1999). Antioxidant activity applying an improved ABTS radical cation decolorization assay. Free Radic Biol Med.

[B36] Rochette L, Guenancia C, Gudjoncik A, Hachet O, Zeller M, Cottin Y, Vergely C (2015). Anthracyclines/trastuzumab: new aspects of cardiotoxicity and molecular mechanisms. Trends Pharmacol Sci.

[B37] Sakanaka S, Tachibana Y, Okada Y (2005). Preparation and antioxidant properties of extracts of Japanese persimmon leaf tea (kakinoha-cha). Food Chem.

[B38] Sandamali J, Hewawasam R, Jayatilaka K, Mudduwa L (2019). Dose dependent cardiac effects of doxorubicin in Wistar rats: A biochemical and histopathological analysis. Int J Pharm Sci Res.

[B39] Satyam SM, Bairy LK, Shetty P, Sainath P, Bharati S, Ahmed AZ, Singh VK, Ashwal A (2023). Metformin and Dapagliflozin attenuate doxorubicin-induced acute cardiotoxicity in Wistar rats: An electrocardiographic, biochemical, and histopathological approach. Cardiovasc Toxicol.

[B40] Shaban NZ, Zaki MM, Koutb F, Abdul-Aziz AA, Elshehawy AA-H, Mehany H (2022). Protective and therapeutic role of mango pulp and eprosartan drug and their anti-synergistic effects against thioacetamide-induced hepatotoxicity in male rats. Environ Sci Pollut Res.

[B41] Sharma A, Parikh M, Shah H, Gandhi T (2020). Modulation of Nrf2 by quercetin in doxorubicin-treated rats. Heliyon.

[B42] Singal PK, Siveski-Iliskovic N, Hill M, Thomas TP, Li T (1995). Combination therapy with probucol prevents adriamycin-induced cardiomyopathy. J Mol Cell Cardiol.

[B43] Singleton VL, Rossi JA (1965). Colorimetry of total phenolics with phosphomolybdic-phosphotungstic acid reagents. Am J Enol Viticult.

[B44] Suzuki H, Suzuki K (1998). Rat hypoplastic kidney (hpk/hpk) induces renal anemia, hyperparathyroidism, and osteodystrophy at the end stage of renal failure. J Vet Med Sci.

[B45] Tan G, Lou Z, Liao W, Zhu Z, Dong X, Zhang W, Li W, Chai Y (2011). Potential biomarkers in mouse myocardium of doxorubicin-induced cardiomyopathy: a metabonomic method and its application. PLoS One.

[B46] Wagh SS, Patil KR, Mahajan UB, Bagal PD, Wadkar AR, Bommanhalli B, Patil PR, Goyal SN, Ojha S, Patil CR (2022). Phloretin-induced suppression of oxidative and nitrosative stress attenuates doxorubicin-induced cardiotoxicity in rats. Asian Pac J Trop Bio.

[B47] Wang J, Yao L, Wu X, Guo Q, Sun S, Li J, Li J, Shi G, Caldwell RB, Caldwell RW, Chen Y (2021). Protection against Doxorubicin-induced cardiotoxicity through modulating iNOS/ARG 2 balance by electroacupuncture at PC6. Oxid Med Cell Longev.

[B48] Yin X, Wu H, Chen Y, Kang YJ (1998). Induction of antioxidants by adriamycin in mouse heart. Biochem Pharmacol.

[B49] Zabihi NA, Mahmoudabady M, Soukhtanloo M, Hayatdavoudi P, Beheshti F, Niazmand S (2018). Salix alba attenuated oxidative stress in the heart and kidney of hypercholesterolemic rabbits. Avicenna J Phytomed.

[B50] Zhang S, You ZQ, Yang L, Li LL, Wu YP, Gu LQ, Xin YF (2019). Protective effect of Shenmai injection on doxorubicin-induced cardiotoxicity via regulation of inflammatory mediators. BMC Complement Altern Med.

[B51] Zhang WB, Zheng YF, Wu YG (2021). Protective effects of Oroxylin A against doxorubicin-induced cardiotoxicity via the activation of Sirt1 in mice. Oxid Med Cell Longev.

[B52] Zhou P, Gao G, Zhao CC, Li JY, Peng JF, Wang SS, Song R, Shi H, Wang L (2022). In vivo and in vitro protective effects of Shengmai injection against doxorubicin-induced cardiotoxicity. Pharm Biol.

